# Predictors of cardiac disease in duchenne muscular dystrophy: a systematic review and evidence grading

**DOI:** 10.1186/s13023-024-03372-x

**Published:** 2024-09-28

**Authors:** Erik Landfeldt, Alberto Alemán, Sophia Abner, Rongrong Zhang, Christian Werner, Ioannis Tomazos, Hanns Lochmüller, Ros M. Quinlivan, Karim Wahbi

**Affiliations:** 1 IQVIA, Pyramidvägen 7, 169 56 Solna, Stockholm, Sweden; 2 Division of Neurology, Department of Pediatrics, Children’s Hospital of Eastern Ontario, Research Institute, University of Ottawa, Ottawa, ON Canada; 3 Division of Neurology, Department of Medicine, The Ottawa Hospital, Brain and Mind Research Institute, University of Ottawa, Ottawa, Canada; 4 IQVIA, London, UK; 5 PTC Therapeutics Sweden AB, Askim, Sweden; 6 PTC Therapeutics Germany GmbH, Frankfurt, Germany; 7 PTC Therapeutics Inc, Warren, NJ USA; 8 Department of Neuropediatrics and Muscle Disorders, Faculty of Medicine and Medical Center, University of Freiburg, Freiburg, Germany; 9 Centre for Neuromuscular Diseases, UCL Institute of Neurology, National Hospital, London, UK; 10 Cardiology Department, AP-HP, Cochin Hospital, Paris, France; 11 Université de Paris, Paris, France

**Keywords:** Heart, Cardiomyopathy, Neuromuscular disease, Treatment, Guidelines, GRADE

## Abstract

**Background:**

Duchenne muscular dystrophy (DMD) is a rare disease that causes progressive muscle degeneration resulting in life-threatening cardiac complications. The objective of this systematic literature review was to describe and grade the published evidence of predictors of cardiac disease in DMD.

**Methods:**

The review encompassed searches of Embase, MEDLINE ALL, and the Cochrane Database of Systematic Reviews from January 1, 2000, to December 31, 2022, for predictors of cardiac disease in DMD. The certainty of evidence (i.e., very low to high) was assessed using the Grading of Recommendations, Assessment, Development and Evaluations (GRADE) framework.

**Results:**

We included 33 publications encompassing 9,232 patients with DMD. We found moderate- to high-quality evidence that cardiac medication (i.e., ACE inhibitors [enalapril and perindopril], β-blockers [carvedilol], and mineralocorticoid receptor antagonists [eplerenone]) are significantly associated with preserved left ventricular ejection fraction (LVEF), left ventricular end-systolic volume (LVESV), and left ventricular circumferential strain (LVCS). *DMD* mutations in exons 51 and 52 were found to be significantly associated with lower risk of cardiomyopathy; deletions treatable by exon 53 skipping and mutations in the Dp116 coding region with improved LVEF and prolonged cardiac dysfunction-free survival; and exons 45–50 and 52 with early left ventricular systolic dysfunction (low/very low-quality evidence). We found high-quality evidence that glucocorticoids (deflazacort) are significantly associated with preserved LVEF and improved fractional shortening (FS), and low-quality evidence that glucocorticoids (deflazacort, prednisone, and/or prednisolone) are associated with improved ejection fraction (EF) and lower risk of cardiomyopathy, ventricular dysfunction, and heart failure-related mortality. Full-time mechanical ventilation was found to be significantly correlated with LVEF (low-quality evidence), muscle strength with FS (low-quality evidence), and genetic modifiers (i.e., *LTBP4 rs10880* and *ACTN3*) with LVEF, lower risk of cardiomyopathy and left ventricular dilation (low-quality evidence).

**Conclusion:**

Several sources of cardiac disease heterogeneity are well-studied in patients with DMD. Yet, the certainty of evidence is generally low, and little is known of the contribution of non-pharmacological interventions, as well as the impact of different criteria for initiation of specific treatments. Our findings help raise awareness of prevailing unmet needs, shape expectations of treatment outcomes, and inform the design of future research.

**Supplementary Information:**

The online version contains supplementary material available at 10.1186/s13023-024-03372-x.

## Background

Duchenne muscular dystrophy (DMD) is a rare, X-linked neuromuscular disease caused by mutations in the *DMD* gene resulting in progressive muscle degeneration, loss of independent ambulation, and life-threatening cardiac and respiratory complications [1]. In the past 50 years, advances in the medical management of DMD have dramatically improved prognosis. Children born in the 1960s seldom survived beyond their second decade of life, which may be compared with recent estimates of life-expectancy of patients receiving current standards of care–including glucocorticoid therapy, spine surgery, and mechanical ventilatory support–of about 30 years [2]. Yet, the unmet medical need and burden of illness remains substantial [3–6].

Following the introduction of the routine use of mechanical ventilatory support in advanced stages of the disease, cardiac involvement has emerged as one of the leading causes of morbidity and mortality in patients with DMD [7]. Dystrophin deficiency in the heart leads to myocardial damage which manifests as cardiomyopathy, resulting in compromised myocardium, potentially fatal rhythm abnormalities, and clinical heart failure. Features of cardiac dysfunction include sinus tachycardia, myocardial fibrosis, and left ventricular enlargement and systolic dysfunction [8]. However, symptoms of cardiac dysfunction (e.g., dyspnea, abdominal pain, fatigue, and inability to perform activities of daily living) are frequently unrecognized in individuals with DMD due to the severe physical impairment associated with the disease, particularly in adults [9]. For that reason, regular follow-up and monitoring is essential to the care strategy of cardiac disease in DMD [8].

Despite their importance for clinical management and prognosis, presently there is a lack of a comprehensive, up-to-date synthesis of predictors of cardiac disease in children and adults with DMD. These include, for example, pharmacological treatments (e.g., glucocorticoids, angiotensin-converting enzyme [ACE] inhibitors, and β-blockers), genetic modifiers associated with dystrophin deficiency and muscle degeneration (e.g., latent TGFβ binding proteins [LTBPs] and the *ACTN3* gene encoding α-actinin-3), and *DMD* mutations [8, 10, 11]. The objective of this systematic literature review was to describe and grade the published evidence of predictors of cardiac disease in DMD.

## Methods

### Search strategy and selection criteria

The bibliographic searches were performed in the following databases: Embase, MEDLINE ALL, and the Cochrane Database of Systematic Reviews. We considered all records published between January 1, 2000 (to ensure relevance to current care practices) and December 31, 2022. We used the search terms “Duchenne muscular dystrophy” as a Medical Subject Heading or free text term, in combination with variations of the term “predictor” (full search strings are provided in eTable 1, eTable 2, and eTable 3 in the Additional file 1). We considered studies of any type, reported in any language, that included male patients diagnosed with DMD exposed to any treatments. We did not consider editorial letters or conference abstracts (as they lack details essential for meaningful synthesis) and did not formally include identified systematic reviews (but screened their reference lists for potential publications). We performed this systematic literature review using guidance from the Preferred Reporting Items for Systematic Reviews and Meta-Analyses (PRISMA) statement [12].

### Screening and data extraction

Screening was conducted independently by two investigators (EL and SA). Conflicts were designated to be resolved by a third reviewer (HL). We extracted the following data elements from included articles: Author; title; study year; geographical setting(s); study design; site(s)/data source(s); study period; sample population characteristics; case ascertainment; pharmacological interventions (incl. number of exposed, dose, and duration of exposure); outcome measures(s); method of analysis; and outcome results. We considered evidence of predictors of cardiac disease, defined as any factor–either endogenous (e.g., *DMD* mutations or genetic modifiers) or exogenous (e.g., pharmacological interventions, including exposure to ACE inhibitors and β-blockers)–significantly associated with cardiac health and function in DMD. We only considered mortality outcomes if the cause of death was established to be related to cardiac involvement. We did not seek to synthesize sources of cardiac variability stemming from cardiac features or assessments (e.g., magnetic resonance imaging or blood biomarkers). Upon identification of the relevant literature, two investigators (EL and SA) systematically screened reference lists of all included publications with the aim to identify additional records of interest not captured by the search strategy.

### Level of evidence

We assessed the certainty of the identified evidence of predictors of cardiac disease in DMD using the Grading of Recommendations, Assessment, Development and Evaluations (GRADE) framework [13]. GRADE rates the overall certainty of evidence based on design limitations, risk of bias, consistency of the results across available studies, the precision of the results, directness, and likelihood of publication. The tool comprises of four levels of evidence, also known as certainty of evidence or quality of evidence: (1) very low (i.e., the true effect is probably markedly different from the estimated effect), (2) low (i.e., the true effect might be markedly different from the estimated effect), (3) moderate (i.e., the authors believe that the true effect is probably close to the estimated effect), and (4) high (i.e., the authors have a lot of confidence that the true effect is similar to the estimated effect). Per the GRADE manual, two investigators (EL and AA) independently provided an initial rating of all included records based on study type. Next, the certainty of evidence at the outcome level was rated down for issues or limitations pertaining to study limitations (e.g., risk of bias due to failure to develop and apply appropriate eligibility criteria, flawed measurement of exposure and/or outcome, failure to adequately control for confounding, and incomplete follow-up), inconsistency of results (i.e., an unexplained heterogeneity of results), imprecision (i.e., a low degree of certainty in reported point estimates), indirectness of evidence (stemming from, for example, differences between populations, differences in interventions, and/or differences in outcome measures), and publication bias (i.e., a systematic under- or over-estimation due to selective publication of studies), and/or rated up in case of a large magnitude of effect, a dose response, or if confounders are likely to minimize the effect. Finally, each investigator independently provided an overall GRADE certainty rating of each outcome and study [13]. All GRADE ratings were subsequently reviewed and confirmed by HL and KW.

## Results

**Fig. 1:**
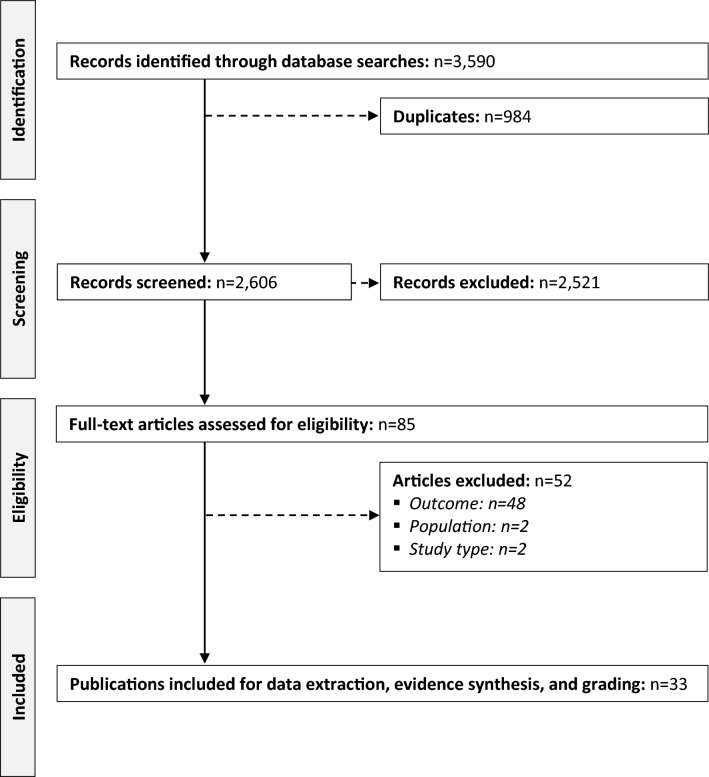
PRISMA diagram of the selection process of the included publications

Upon completion of the bibliographic searches, we identified a total of 3,590 articles, of which 984 were duplicates. After full-text review of 85 records, 33 articles [14–46] were ultimately included. Figure 1 presents the PRISMA flow chart of the study selection process. Identified studies encompassed 9,232 patients with DMD from 11 countries (i.e., Brazil, Canada, China, Germany, France, Greece, Italy, Japan, South Korea, the United Kingdom (UK), and United States of America (US) (two multi-national studies [26, 28] did not explicitly disclose included countries) (Table 1). Yet, as some countries were represented by more than one study each, we cannot rule out that a proportion of patients might have been included more than once. In total, 15% (5 of 33) of articles described results from randomized research, 6% (2 of 33) from prospective cohort studies, 76% (25 of 33) from retrospective cohort studies, and 3% (1 of 33) from case series.

**Table 1:** Characteristics of included studies

Author (year) [country]	Study design	Data source(s)/site(s) and study period	Inclusion/ exclusion criteria	Case ascertainment	Sample, n (age)^†^	Pharmacological intervention(s)	n (%) exposed^†^	Dose, mean	Duration of exposure, mean
Aikawa et al. (2019) [JP] [14]	Retrospective cohort study	Hokkaido University Hospital (Sapporo, JP) 2013–2017	*Inclusion criteria:* Diagnosis of DMD or BMD Referred for clinically indicated CMR imaging *Exclusion criterion:* Diagnosis of other neuromuscular disease	Clinical presentation, genetic testing, muscle biopsy, and family history of DMD or BMD	34 patients with DMD or BMD (median age: 12 years, IQR: 6–16 years)	ACE inhibitors (cilazapril and enalapril)	20 (59%)	*At start of study* Cilazapril: 1.5 (IQR: 1.5–1.5) mg/day^a,b^ Enalapril: NR *At end of study* Cilazapril: 1.5 (IQR: 1.5–1.5) mg/day^a,b^ Enalapril: 10 (IQR: 7.2–10.0) mg/day^a,b^	*At start of study* 1.6 (IQR: 0.8–2.3) years^a,b^ *At end of study* 2.7 (IQR: 1.5–4.2) years^a,b^
						ARBs (agents NR)	1 (3%)	*At start of study* NA *At end of study* NR	*At start of study* NA *At end of study* 1.0 (IQR: 1.0–1.0) years^a^
						β-blockers (bisoprolol)	5 (15%)	*At start of study* NR *At end of study* 2.5 (IQR: 0.625–2.5) mg/day^a,b^	*At start of study* 1.3 (IQR: 0.8–2.3) years^a,b^ *At end of study* 2.8 (IQR: 1.1–5.0) years^a,b^
						Glucocorticoids (PRED)	8 (24%)	*At start of study* 7.0 (IQR: 5.0–8.3) mg/day^a.b^ *At end of study* NR	*At start of study* 1.0 (IQR: 0.5–3.0) years^a,b^ *At end of study* 3.7 (IQR: 3.0–5.2) years^a,b^
Barber et al. (2013) [US] [15]	Retrospective cohort study	MD STAR*net* (US, multi-centre) 1982–2010	*Inclusion criterion:* Diagnosis of DMD *Exclusion criteria:* Cardiac function could not be determined through echocardiographic records Glucocorticoid treatment could not be classified	NR^c^	462 patients with DMD (mean age: NR, range: NR)	Glucocorticoids (DFZ, PDN, or PRED)	291 (63%)	NR	4.1 (SD: 3.4) years
Barp et al. (2015) [IT] [16]	Retrospective cohort study	Universities of Padova, Naples, Messina, and Milan (IT); and NEuroMuscular Omnicenter (Milan, IT) Study period NR	*Inclusion criteria:* Diagnosis of DMD Record of a regular (annual) cardiologic follow-up (incl. 2D-M-mode echocardiography) Availability of a DNA sample	Genetic testing and/or muscle biopsy	178 patients with DMD (mean age: NR, range: NR)	ACE inhibitors (cilazapril and enalapril)	0 (0%)	NA	NA
						ARBs (agents NR)	0 (0%)	NA	NA
						β-blockers (bisoprolol)	0 (0%)	NA	NA
						Glucocorticoids (DFZ or PDN)	75 (42%)	DFZ: 0.9 mg/kg/day PDN: 0.75 mg/kg/day	NR
Batra et al. (2022) [US] [17]	Prospective cohort study	University of Florida (Gainesville, US); and University of California Davis (Davis, US) Study period NR	*Inclusion criterion:* Diagnosis of DMD	Genetic testing and/or muscle biopsy	59 patients with DMD (mean age: 12 years, range: 5–18 years)	Glucocorticoids (agents NR)	52 (88%)	NR	NR
						ACE inhibitors (agents NR) and/or ARBs (agents NR)	35 (59%)	NR	NR
Biggar et al. (2006) [CA] [18]	Retrospective cohort study	The Bloorview MacMillan Children’s Center (Toronto, CA) 1990–2004	*Inclusion criteria:* Diagnosis of DMD 10–18 years of age Could cooperate for reproducible muscle and pulmonary function testing	Age at onset of symptoms (< 5 years of age), male sex, proximal muscle weakness, increased serum creatine kinase levels, and muscle biopsy and/or genetic testing	74 patients with DMD (mean age: 15 years, range: 10–18 years)	Glucocorticoids (DFZ)	40 (54%)	*Initial dose* 0.9 mg/kg/day *At 10 years of age* 0.8 (0.18) mg/kg/day *At 15 years of age* 0.55 (0.09) mg/kg/day *At 18 years of age* 0.5 (0.2) mg/kg/day	5.5 years
Cirino et al. (2018) [BR] [19]	Retrospective cohort study	The Neuromuscular Disorders Service of the Hospital de Clíınicas of the Federal University of Parana (Curitiba, BR) 2014–2016	*Inclusion criterion:* Diagnosis of DMD *Exclusion criteria:* Unable to be present for the prescheduled medical appointment or the echocardiography examination Suboptimal echocardiographic imaging that prevented proper evaluation of LVSF Overt LVSD (LVEF < 52%)	Clinical presentation, and genetic testing and/or muscle biopsy	40 patients with DMD (mean age: 11 years, range: 2–19 years)	Glucocorticoids (agents NR)	31 (78%)	NR	3.5 years^d^
Dittrich et al. (2019) [DE] [20]	RCT	DE, multi-centre 2010–2015	*Inclusion criteria:* Diagnosis of DMD 10–14 years of age Preserved LV function as defined by echocardiography with LVFS ≥ 30% in the long-axis motion-mode Normal renal function with glomerular filtration rate > 30 ml/min/1.73 m^2^ Ability to participate in the assessment of primary and secondary outcome measures *Exclusion criteria:* Contraindication for treatment with ACE inhibitors or β-blockers Previous treatment with ACE inhibitors or β-blockers in the past 3 months Abnormal liver function defined by elevation (≥ 2x) of gamma-glutamyltranspeptidase and bilirubine LV dilation above the 97th percentile as defined by echocardiography in the long-axis motion-mode Participation in other clinical trials	Genetic testing and/or muscle biopsy	38 patients with DMD (mean age: 11 years, range: 9–13 years)	ACE inhibitors (enalapril) and β-blockers (metoprolol)	21 (55%)	Study phase-specific (see article for details)	Study phase-specific (see article for details)
						Glucocorticoids (agents NR)	*Current or historical use* 26 (68%) *Use during follow-up* 21 (55%)	NR	NR
Duboc et al. (2005) [FR] [21]	RCT and open-label extension	FR, multi-centre Study period NR	*Inclusion criteria:* Diagnosis of DMD Age 9.5–13 years Normal cardiac examination Radionuclide LVEF > 55% Tolerate a 1-mg test dose of perindopril Systolic blood pressure ≥ 80 mm Hg in the supine or > 70 mm Hg in the sitting position *Exclusion criteria:* Patients treated with cardioactive drugs Blood urea nitrogen > 7 mmol/l Contraindications to ACE inhibitor therapy	Genetic testing	57 patients with DMD (mean age: 11 years, range: 9–13 years)	ACE inhibitors (perindopril)	28 (49%)	2–4 mg/day	60 months
						β-blockers (agents NR)	9 (18%)	NR	NR
Fayssoil et al. (2018) [FR] [22]	Retrospective cohort study	The Home Mechanical Ventilation Unit of the Raymond Poincare University Hospital (Garches, France) 2006–2016	*Inclusion criteria:* Diagnosis of DMD Annual cardiopulmonary evaluation and diaphragm ultrasound *Exclusion criterion:* Full-time invasive ventilation support	NR	101 adult patients with DMD (median age: 21 years, IQR: 18–26 years)	ACE inhibitors (agents NR)	97 (87%)	NR	NR
						β-blockers (agents NR)	58 (52%)	NR	NR
						Diuretics (agents NR)	12 (11%)	NR	NR
						Glucocorticoids (agents NR)	0 (0%)	NA	NA
Houde et al. (2008) [CA] [23]	Retrospective cohort study	The multidisciplinary Neuromuscular Clinic of the Marie-Enfant Rehabilitation Centre (Montreal, CA) Study period NR	*Inclusion criterion:* Diagnosis of DMD *Exclusion criterion:* Unable to participate in segmental muscle testing or pulmonary function tests	Genetic testing and/or muscle biopsy	79 patients with DMD (mean age: 11 years, range: NR)	Glucocorticoids (DFZ)	37 (47%)	*Initial dose* 0.9 mg/kg per day (adjusted according to evolution or side effects to a maximum of 1 mg/kg) *At last visit* 0.69 (SD: 0.20) mg/kg	66 months
						ACE inhibitors (agents NR)	30 (38%)	NR	NR
Jefferies et al. (2005) [US] [24]	Retrospective cohort study	The Texas Children’s Hospital Cardiovascular Genetics Clinic (Houston, US) Study period NR	*Inclusion criterion:* Diagnosis of DMD or BMD	Genetic testing	69 patients with DMD and BMD (mean age: 15 years, range: NR)	ACE inhibitors (enalapril, captopril, and lisinopril)	31 (45%)	Enalapril: 3.6 mg (range: 2.5–10 mg) twice daily Captopril: 7.6 mg (range: 6–10 mg) three times daily Lisinopril: 5 mg/day (range: NR)	2.7 years
						β-blockers (carvedilol and metoprolol)	18 (26%)	Metoprolol: 21 mg (range: 5–50 mg) twice daily Carvedilol: 4 mg (range: 3.125–6.25 mg) twice daily	1.6 years
Kajimoto et al. (2006) [JP] [25]	Retrospective cohort study	Data source(s)/site(s NR 1999–2002	*Inclusion criterion:* Diagnosis of DMD, FCMD, or EDMD	NR	28 patients with DMD, FCMD, or EDMD (mean age: 17 years, range: 7–29 years)	β-blockers (carvedilol) (administered in combination with ACE inhibitors)	13 (46%)	*Initial dose* 0.01–0.02 mg/kg (maximum: 1 mg) twice daily *At end of study* 0.5–1 mg/kg (maximum: 20 mg)	2 years
						ACE inhibitors (cilazapril or enalapril)	28 (100%)	*At end of study* Cilazapril: 0.03 mg/kg/day Enalapril: 0.3 mg/kg/day	3 years
						Diuretics (furosemide or spironolactone)	NR	NR	NR
Kelley et al. (2022) [*] [26]	Retrospective cohort study	CINRG DNHS (multi-country, multi-centre) Study period NR	NR^c^	Genetic testing	147 patients with DMD (mean age: 12 years, range: 4–30 years)	Glucocorticoids (agents NR)	125 (85%)	NR	7.71 years
						β-blockers (agents NR)	29 (20%)	NR	NR
						ACE inhibitors (agents NR) and/or ARBs (agents NR)	93 (63%)	NR	NR
						Diuretics (agents NR)	18 (12%)	NR	NR
						Anti-arrhythmics (agents NR)	35 (24%)	NR	NR
						Inotropes (agents NR)	7 (5%)	NR	NR
Kim et al. (2017) [US] [27]	Retrospective cohort study	MD STAR*net* (US, multi-centre) 1982–2011	*Inclusion criteria:* Diagnosis of DMD ≥ 1 year of follow-up	Age at onset of symptoms (≤ 5 years of age), male sex, clinical presentation, genetic testing, muscle biopsy, increased serum creatine kinase levels, and/or family history (see article for further details)	660 patients with DMD (mean age: NR, range: NR)	Glucocorticoids (DFZ, PDN, or PRED)	318 (48%)	NR	5.9–6.4 years
Koeks et al. (2017) [*] [28]	Retrospective cohort study	The TREAT-NMD global DMD database (multi-national, multi-centre) 2007–2013	*Inclusion criterion:* Diagnosis of DMD	Genetic testing	5,345 patients with DMD (mean age: NR, range: NR)	Glucocorticoids (DFZ, PDN, or PRED)	*Current use* 2,658 (50%) *Past use* 522 (10%)	NR	NR
Kwon et al. (2012) [KR] [29]	Randomised trial	Seoul National University Children’s Hospital (Seoul, KR) 2008–2011	NR	NR	23 patients with DMD or BMD (mean age: 13 years, range: NR)	ACE inhibitors (enalapril)	13 (57%)	*Initial dose* 0.05 mg/kg/day *At end of study* 0.1 mg/kg/day	21 (SD: 11) months
						β-blockers (carvedilol)	10 (43%)	*Initial dose* 0.075 mg/kg every 12 h *At end of study* 1 mg/kg/day	19 (SD: 7) months
Markham et al. (2005) [US] [31]	Retrospective cohort study	Data source(s)/site(s NR 1997–2004	*Inclusion criteria:* Diagnosis of DMD < 22 years of age Complete medical records (incl. echocardiographic and glucocorticoid data)	NR	111 patients with DMD (mean age: 12 years, range: 3–21 years)	Glucocorticoids (DFZ or PDN)	48 (43%)	NR	3 (SD: 3) years
Markham et al. (2008) [US] [30]	Retrospective cohort study	Data source(s)/site(s NR 1998–2006	*Inclusion criteria:* Diagnosis of DMD < 9 years of age Glucocorticoid-naive at initial cardiac evaluation ≥ 3 complete echocardiographic studies, each at least 1 year apart Complete medical records for history of glucocorticoid exposure	Clinical presentation and genetic testing and/or muscle biopsy	37 patients with DMD (mean age: 8 years, range: NR)	Glucocorticoids (DFZ or PDN)	14 (38%)	DFZ: 0.9 mg/kg/day PDN: 0.75 mg/kg/day	NR
Matsumura et al. (2010) [JP] [32]	Retrospective cohort study	JP, multi-centre Study period NR	*Inclusion criterion:* Diagnosis of DMD *Exclusion criteria:* Acute heart failure, active asthma, severe uncontrolled arrhythmia, and/or advanced atrioventricular block Therapy with agents influencing the sympathetic nervous system Bradycardia < 50 BPM Systolic blood pressure < 80 mm Hg Severe structural deformity precluding ultrasound cardiography Contraindications to ACE inhibitors, ARBs, or β-blockers	NR	54 patients with DMD (mean age: 20 years, range: 11–35 years)	β-blockers (carvedilol)	41 (76%)	*Initial dose* 0.3125 mg or 0.625 mg twice daily *At end of study* 10 mg/day (range: 5–20 mg/day)	1,385 (SD: 470) days
						ACE inhibitors (agents NR) or ARBs (agents NR)	13 (24%)	NR	1,444 (608) days
						Pimobendan	9 (17%)	1.25–5.0 mg/day	NR
						Diuretics (agents NR)	NR	NR	NR
Mavrogeni et al. (2009) [GR] [33]	Prospective cohort study	Data source(s)/site(s NR Study period NR	*Inclusion criteria:* Diagnosis of DMD Treated with deflazacort for ≥ 7 years (treated group) or no glucocorticoid treatment (untreated group)	Genetic testing	34 patients with DMD (mean age: NR, range 12–22 years)	Glucocorticoids (DFZ)	17 (50%)	0.9 mg/kg/day	7–14 years
Nagai et al. (2020) [JP] [34]	Retrospective cohort study	The Department of Pediatrics, Kobe University Hospital (Kobe, JP) 1992–2018	*Inclusion criteria:* Diagnosis of DMD Available genomic DNA Routine echocardiography examinations No echocardiographic findings indicating cardiac dysfunction or dilated cardiomyopathy at the first examination	Genetic testing	77 patients with DMD (median age: 9 years, IQR: 8–12 years)	Glucocorticoids (PDN)	29 (28%)	NR	NR
						ACE inhibitors (agents NR)	36 (47%)	NR	NR
						β-blockers (agents NR)	31 (40%)	NR	NR
Porcher et al. (2021) [35]	Retrospective cohort study	The French multicentre DMD Heart Registry (FR, multi-centre) (NCT03443115) 1986–2018	*Inclusion criterion:* Diagnosis of DMD *Exclusion criteria:* Diagnosed with DMD before January 1986 Concurrent illness that could also cause cardiac or respiratory disease or influence the vital prognosis Unknown date of treatment initiation	Clinical presentation and genetic testing	576 patients with DMD (mean age: 6 years, range: NR)	Glucocorticoids (DFZ, PDN, or PRED)	*At baseline* 18 (3%) *At end of study* 178 (31%)	NR	NR
						ACE inhibitors (perindopril, enalapril, ramipril, or lisinopril)	*At baseline* 0 (0%) *At end of study* 390 (68%)	NR	NR
						β-blockers (bisoprolol, nebivolol, carvedilol, atenolol, propranolol, or nadolol)	*At baseline* 3 (< 1%) *At end of study* 100 (17%)	NR	NR
						Mineralocorticoid receptor antagonist (spironolactone or eplerenone)	*At baseline* 1 (< 1%) *At end of study* 31 (5%)	NR	NR
Posner et al. (2016) [US] [36]	Retrospective cohort study	Vanderbilt University Medical Center (Nashville, US) 1995–2013	*Inclusion criterion:* Diagnosis of DMD *Exclusion criteria:* Unclear neuromuscular diagnosis or diagnosis of other neuromuscular disease No echocardiogram performed or no objective measures of LV function that could be paired with measures of skeletal muscle function	Clinical presentation and genetic testing and/or muscle biopsy	77 patients with DMD (mean age: 14 years, range: 3–35 years)	Glucocorticoids (agents NR)	60 (78%)^e^	NR	3.4 (SD: 2.5) years
						ACE inhibitors (agents NR)	37 (48%)^e^	NR	2.9 (SD: 2.9) years
						ARBs (agents BR)	9 (12%)^e^	NR	3.9 (SD: 1.8) years
						β-blockers (agents NR)	23 (30%)^e^	NR	3.3 (SD: 2.0) years
						Mineralocorticoid receptor antagonists (agents NR)	3 (4%)^e^	NR	2.0 (SD: 1.8) years
Raman et al. (2015) [US] [37]	RCT	US, multi-centre 2012–2013	*Inclusion criteria:* Diagnosis of DMD ≥ 7 years of age Myocardial damage in one or more LV segments evident by late gadolinium enhancement Preserved LVSF, defined as LVEF ≥ 45% by cine cardiac MRI Background ACE inhibitors or ARB therapy (selection of which was dictated by clinical care) *Exclusion criteria:* MRI-incompatible implants Severe claustrophobia Allergy to gadolinium contrast Previous use of eplerenone or spironolactone Use of potassium-sparing diuretics Use of another investigational agent within 4 weeks or five half-lives of the drug, whichever was longer, before screening Scheduled surgery that would increase the risks of or potentially result in adverse events Use of CYP3A4 strong inhibitors	Clinical presentation and/or genetic testing	42 patients with DMD (median age: 15 years, IQR: NR)	Mineralocorticoid receptor antagonists (eplerenone)	20 (48%)	25 mg every other day (increased to once daily if tolerated)	NR
						ACE inhibitors (agents NR)	38 (90%)	NR	NR^f^
						ARBs (agents NR)	4 (10%)	NR	NR^f^
						β-blockers (agents NR)	17 40%)	NR	1.5 years
						Glucocorticoids (DFZ or PDN)	35 (83%)	DFZ: 25 mg/day PDN: 30 mg/day	5.4 years
						Loop diuretics (furosemide)	2 (5%	NR	NR
Schram et al. (2013) [CA] [38]	Retrospective cohort study	The Neuromuscular Clinic of the Marie-Enfant Rehabilitation Center, Université de Montréal (Montréal, CA) 1972–2006	*Inclusion criterion:* Diagnosis of DMD	Genetic testing or muscle biopsy	86 patients with DMD (mean age: 9 years, range: NR)	Glucocorticoids (DFZ or PDN)	63 (73%)	DFZ: 0.9 mg/kg/day PDN: 0.5–0.75 mg/kg/day	NR
						ACE inhibitors (agents NR)	74 (86%)	NR	NR
						ARBs (agents NR)	35 (41%)	NR	NR
						β-blockers (agents NR)	53 (62%)	NR	NR
						Cardiotonic agents (digoxin)	22 (26%)	NR	NR
						Diuretics (agents NR)	8 (9%)	NR	NR
Servais et al. (2015) [FR] [39]	Case series	Pre-U7 (multi-country, multi-centre [NCT01385917]); and ULENAP (FR, multi-centre [NCT00993161]) Study period NR^c^	*Pre-U7 inclusion criteria:* Diagnosis of DMD theoretically treatable by exon 53 skipping 6–20 years of age Able to understand rules of assessments Signed informed consent *ULENAP inclusion criteria:* Diagnosis of DMD 5–30 years of age Able to understand rules of assessments Non-ambulant Signed informed consent	NR^c^	35 patients with DMD (mean age: 14 years, range: NR)	Glucocorticoids (agents NR)	6 (17%)	20 mg/day	NR
						ACE inhibitors (agents NR)	24 (69%)	NR	NR
Silva et al. (2017) [BR] [40]	RCT	Federal University of Minas Gerais (Belo Horizonte, Brazil); and Heart Institute, InCor, University of São Paulo (São Paulo Brazil) 2009–2012	*Inclusion criteria:* Diagnosis of DMD ≥ 6 years of age	Genetic testing and/or muscle biopsy	42 patients with DMD or BMD (mean age: 12 years, range: NR)	Glucocorticoids (agents NR)	52 (68%)	0.75 mg/kg/day (10 days on, 10 days off)	2 years
						ACE inhibitors (enalapril)	21 (50%)	< *10 years of age* 2.5 mg every 12 h (maximum: 5 mg) *10–15 years of age* 5 mg every 12 h (maximum: 10 mg) > *15 years of age* 10–20 mg every 12 h	2 years
Silversides et al. (2003) [CA] [41]	Retrospective cohort study	The Bloorview MacMillan Children’s Center (Toronto, CA) 1998–2002	*Inclusion criteria:* Diagnosis of DMD 10–18 years of age	Age at onset of symptoms (< 5 years of age), male sex, proximal muscle weakness, increased serum creatine kinase levels, and muscle biopsy and/or genetic testing	33 patents with DMD (mean age: 15 years, range: 10–18 years)	Glucocorticoids (DFZ)	21 (64%)	*Initial dose* 0.9 mg/kg/day	5.1 (SD: 2.4) years
						ACE inhibitors (agents NR)	6 (18%)	NR	NR
						Cardiotonic agents (digoxin)	3 (9%)	NR	NR
Tandon et al. (2015) [US] [42]	Retrospective cohort study	Cincinnati Children’s Hospital Medical Center (Cincinnati, US) 2005–2013	*Inclusion criteria:* Diagnosis of DMD ≥ 4 clinical CMR studies in which LGE status could be determined	NR	98 patients with DMD (mean age: 13 years, range: 7–29 years)	Glucocorticoids (DFZ and/or PDN)	95 (97%)	NR	7.6 (SD: 3.4) years
						ACE inhibitors (agents NR)	NR	NR	NR
						β-blockers (agents NR)	NR	NR	NR
Trucco et al. (2020) [UK] [43]	Retrospective cohort study	Dubowitz Neuromuscular Centre (London, UK) 2000–2017	*Inclusion criteria:* Diagnosis of DMD < 18 years of age *Exclusion criteria:* Enrolment in the Heart Protection Trial Enrolment in any interventional clinical trials	NR	270 patients with DMD (mean age: 6 years, range: NR)	Glucocorticoids (DFZ or PDN)	248 (92%)	NR	NR
						ACE inhibitors (agents NA)	0 (0%)	NA	NA
						ARBs (agents NA)	0 (0%)	NA	NA
						β-blockers (agents NA)	0 (0%)	NA	NA
						Cardiotonic agents (agents NR)	0 (0%)	NA	NA
						Diuretics (agents NR)	0 (0%)	NA	NA
						Mineralocorticoid receptor antagonists (agents NA)	0 (0%)	NA	NA
Viollet et al. (2012) [US] [44]	Retrospective cohort study	The Nationwide Children’s Hospital (Columbus, US) 2005–2011	*Inclusion criterion:* Diagnosis of DMD *Exclusion criteria:* Therapy with an ACE inhibitor exceeded 6 months’ duration before initial evaluation Poor echocardiographic images precluded accurate evaluation of ejection fraction No pretherapy data < 6 months follow-up data after starting an ACE inhibitor	Genetic testing or muscle biopsy	42 patients with DMD (mean age: 14 years, range: 7–27 years)	ACE inhibitors (lisinopril)	42 (100%)	*Initial dose* 0.09 (SD: 0.03) (range: 0.03–0.17) mg/kg/day *At end of study* 0.16 (SD: 0.1) (range: 0.06–0.49) mg/kg/day	NR
						β-blockers (metoprolol or atenolol)	24 (57%)	*Initial dose* Metoprolol: 1–2 mg/kg Atenolol: NR *At end of study* Metoprolol: 1.1 (SD: 0.6) mg/kg Atenolol: NR	19 months
						Glucocorticoids (DFZ or PDN)	13 (31%)	NR	NR
Yamamoto et al. (2018) [JP] [45]	Retrospective cohort study	Department of Pediatrics, Kobe University Hospital (Kobe, JP) 2007–2017	*Inclusion criterion:* Diagnosis of DMD	Genetic testing	181 patients with DMD (mean age: 10 years, range: 4–25 years)	ACE inhibitors (agents NR)	93 (51%)	NR	NR
						β-blockers (agents NR)	92 (51%)	NR	NR
						Glucocorticoids (agents NR)	80 (44%)	NR	NR
Zhang et al. (2015) [CN] [46]	Retrospective cohort study	The Neuromuscular Disorders Department of the Third Hospital of Hebei Medical University (Hebei, CN) 2008–2012	*Inclusion criterion:* Diagnosis of DMD *Exclusion criteria:* Valvular heart disease LV hypertrophy Other systemic diseases	Clinical presentation, creatine kinase levels, and muscle biopsy	43 patients with DMD (mean age: 8 years, range: 7–10 years)	Glucocorticoids (agents NR)	43 (100%)	NR	2 years

Angiotensin-converting enzyme (ACE). Angiotensin receptor blocker (ARB). Beats per minutes (BPM). Becker muscular dystrophy (BMD). Brazil (BR). Canada (CA). China (CN). Cardiovascular magnetic resonance (CMR). Cooperative International Neuromuscular Research Group Duchenne Natural History Study (CINRG-DNHS). Deflazacort (DFZ). Deoxyribonucleic acid (DNA). Duchenne muscular dystrophy (DMD). Emery-Dreifuss muscular dystrophy (EDMD). France (FR). Fukuyama type congenital muscular dystrophy (FCMD). Greece (GR). Inter-quartile range (IQR). Japan (JP). Left ventricular (LV). Left ventricular ejection fraction (LVEF). Left ventricular fractional shortening (LVFS). Left ventricular systolic dysfunction (LVSD). Left ventricular systolic function (LVSF). Magnetic resonance imaging (MRI). Muscular Dystrophy Surveillance, Tracking, and Research Network (MD STARnet). Not applicable (NA). Not reported (NR). Prednisolone (PRED). Prednisone (PDN). Randomized controlled trial (RCT). South Korea (KR). Standard deviation (SD). United States of America (US)

^†^Details for the sample analysed with respect to outcomes of cardiac disease

^*^Multi-national (see article for details)

^a^In patients with DMD (n = 21 at start of study and n = 14 at end of study)

^b^Median

^c^Details not reported but provided in referenced publications

^d^The mean time of glucocorticoid treatment was 2.0 (SD: 2.3) years in patients without left ventricular systolic dysfunction and 5.0 (SD: 3.0) years in patients with early left ventricular systolic dysfunction

^e^Current or previous exposure

^f^Duration of exposure was 1.6 years for ACE inhibitors and ARBs

### Predictors of cardiac disease in DMD

#### Cardiac medication

We identified three randomized controlled trials (RCTs) reporting evidence of benefits of cardiac medication on left ventricular ejection fraction (LVEF) in patients with DMD (Table 2). Specifically, in the RCT and open-label extension by Duboc et al. [21], encompassing 57 French children with DMD (mean age: 11 years; range: 9–13), the proportion with LVEF < 45% after 60 months of follow-up was significantly lower among those treated with ACE inhibitors (perindopril) (initiated at a LVEF > 55%), in some cases administered in combination with β-blockers (agents not reported), compared with no ACE inhibitor treatment (4% vs. 28%, p = 0.02). Similarly, in the RCT by Silva et al. [40], treatment with ACE inhibitors (enalapril) (initiated at a LVEF > 50%) was found to be associated with slower myocardial fibrosis (MF) progression identified on cardiovascular magnetic resonance among 42 Brazilian patients (39 with DMD and three with Becker muscular dystrophy [BMD], a milder allelic condition also caused by in-frame mutations in the *DMD* gene; mean age: 12 years, range not reported) across 24 months of follow-up. Lastly, in the RCT by Raman et al. [37], involving 42 US participants with DMD (median age: 15 years, inter-quartile range [IQR] not reported), those treated with mineralocorticoid receptor antagonists (eplerenone) (initiated at a LVEF > 45%) were found to have significantly lower decline in LVEF after 12 months of follow-up (median change from baseline: -1.8% [treated] vs. -3.7% [untreated], p = 0.032). However, some cases were concurrently receiving ACE inhibitors (agents not reported), angiotensin receptor blockers (ARBs) (agents not reported), β-blockers (agents not reported), and/or loop diuretics (furosemide). Significant differences were also noted for left ventricular end-systolic volume (LVESV) (median change from baseline: -1.64 ml [treated] vs. 4.07 ml [untreated], p = 0.034), as well as left ventricular circumferential strain (LVCS) (median change from baseline: 1.0% [treated] vs. 2.2% [untreated], p = 0.020). Additionally, we identified one RCT reporting evidence of benefits of cardiac medication on heart rate (HR), PQ-interval, and P-wave in patients with DMD. Specifically, Dittrich et al. [20] examined the effects of combined treatment with ACE inhibitors (enalapril) and β-blockers (metoprolol) in a German cohort encompassing 38 children with DMD (mean age: 11 years, range: 9–13). At 19 months after randomization, patients receiving enalapril and metoprolol (initiated at a left ventricular fractional shortening [LVFS] ≥ 30%) were found to have significantly improved HR, P-wave, and PQ-interval compared with those treated with placebo (all p < 0.05).

**Table 2:** GRADE assessment of studies of predictors of cardiac disease in DMD

Author (year) [country]	Predictor(s)/indicator(s)	Outcome measure(s)	Method of analysis	Outcome results	Initial GRADE	Outcome GRADE modification	Overall GRADE
Aikawa et al. (2019) [JP] [14]	ACE inhibitors (cilazapril and enalapril)	LVEF (%)	Regression analysis (mixed-effects model)	β (treatment vs. no treatment): -3.1, 95% CI: -0.8 to -5.4, p = 0.009 β (treatment at LVEF < 55% vs. no treatment): 3.7, 95% CI: 0.9 to 6.4, p = 0.009	Low	Very low (indirectness; estimates for DMD and BMD)	Very low
Barber et al. (2013) [US] [15]	Glucocorticoids (DFZ, PDN, or PRED)	Cardiomyopathy (FS < 28% or EF < 55%)^a^	Kaplan–Meier (log-rank test)	Survival functions (treated vs. untreated): p = 0.02	Low	–	Low
			Regression analysis (accelerated failure time survival model, distribution NR)	β (treatment duration): 0.04, SE: 0.07, 95% CI: 0.026 to 0.054, p < 0.001		–	
Barp et al. (2015) [IT] [16]	Genotype LTBP4 rs10880 (CC, CT, and TT)	DCM (LVEDV > 70 ml/m^2^ and/or LVEF < 50%)	Kaplan–Meier (log-rank test)	*Median age at DCM (glucocorticoid-treated patients):* 17.9 (CC/CT) vs. NA (< 50% DCM) (TT), p < 0.027	Low	–	Low
Batra et al. (2022) [US] [17]	ACE inhibitors (agents NR) and/or ARBs (agents NR)	LVM (gm)	Regression analysis (Bayesian linear model)	β (treatment vs. no treatment): -5.6, p < 0.05	Low	–	Low
		LVESV (ml)		β (treatment vs. no treatment): -2.6, p < 0.05		–	
		LVEDV (ml)		β (treatment vs. no treatment): -5.9, p < 0.05		–	
Biggar et al. (2006) [CA] [18]	Glucocorticoid (DFZ)	LVEF (%)	Descriptive (Fisher’s exact test)	*Proportion with LVEF* < *45% at 18 years of age* 10% (4 of 40) (treated) vs. 58% (20 of 34) (untreated), p < 0.001	High	–	High
		FS (%)	Descriptive (Student’s *t*-test)	*Mean (SD) at 18 years of age* 33% (7%) (treated) vs. 21% (8%) (untreated), p < 0.002		–	
Cirino et al. (2018) [BR] [19]	Exon mutation (1 to 79)	Left ventricular systolic dysfunction	Descriptive (Fisher’s exact test)	Exons 45, 46, 47, 48, 49, 50, and 52 were associated with early left ventricular systolic dysfunction (all p < 0.044)	Low	Very low (small sample size)	Very low
Dittrich et al. (2019) [X] [20]	ACE inhibitors (enalapril) and β-blockers (metoprolol)	P-wave (ms)	Regression analysis (mixed-effects model)	*Mean (95% CI) adjusted difference (treated vs untreated) 19 months after randomization* 10.3 (2.1 to 18.6), p < 0.05	High	Moderate (inconsistency of results)	Moderate
		PQ-interval (ms)		*Mean (95% CI) adjusted difference (treated vs untreated) 19 months after randomization* 10.9 (2.1 to 19.7), p < 0.05			
		HR (bpm)		*Mean (95% CI) adjusted difference (treated vs untreated) 19 months after randomization* -16.7 (-25.6 to − 7.9), p < 0.05			
Duboc et al. (2005) [FR] [21]	ACE inhibitors (perindopril)	LVEF (%)	Descriptive (χ^2^ test)	*Proportion with LVEF* < *45% at end of study* 4% (1 of 28) (treated) vs. 28% (8 of 29) (untreated), p = 0.02	High	Moderate (inconsistency of results)	Moderate
Fayssoil et al. (2018) [FR] [22]	Mechanical ventilation	LVEF (%)	Correlation analysis (Spearman’s correlation coefficient [ρ])	Correlation between duration of full-time mechanical ventilation (per diem) and annual change in LVEF: ρ: -0.31, p = 0.012	Low	–	Low
Houde et al. (2008) [CA] [23]	Glucocorticoid (DFZ)	FS (%)	Descriptive (Student’s *t*-test)	*Mean (SD) at end of follow-up* 30.8% (4.5%) (treated) vs. 26.6% (5.7%) (untreated), p < 0.05	Low	–	Low
		EF (%)		*Mean (SD) at end of follow-up* 52.9% (6.3%) (treated) vs. 46.0% (10%) (untreated), p < 0.05		–	
		DCM (FS < 28% or LVEDD > 95th percentile)		*Proportion with DCM at end of study* 32% (12 of 38) (treated) vs. 58% (28 of 48) (untreated), p < 0.05		–	
Jefferies et al. (2005) [US] [24]	ACE inhibitors (enalapril, captopril, and lisinopril) and/or β-blockers (carvedilol and metoprolol)	LVEDD (cm)	Descriptive (Student’s *t*-test)	*Mean (SD) across follow-up* 5.2 (0.9) cm (baseline) vs. 4.8 (0.9) cm (end of follow-up), p = 0.001	Low	Very low (indirectness; estimates for DMD and BMD)	Very low
		LVEF (%)		*Mean (SD) across follow-up* 36% (11%) (baseline) vs. 53% (12%) (end of follow-up), p < 0.001			
		LV MPI		*Mean (SD) across follow-up* 0.53 (0.2) (baseline) vs. 0.38 (0.1) (end of follow-up), p < 0.001			
		Left ventricular sphericity index		*Mean (SD) across follow-up* 0.73 (0.1) (baseline) vs. 0.59 (0.1) (end of follow-up), p < 0.001			
	Exon mutation (12,14, 15, 16, 17, 51, and 52)	Cardiomyopathy (EF < 55% or LV dilation^b^)	Correlation analysis (Fisher’s exact test)	*Association between DMD mutations and age at onset of cardiomyopathy* Exon 12 (p = 0.03)^c^ Exon 14 (p = 0.01)^c^ Exon 15 (p = 0.03)^c^ Exon 16 (p = 0.03)^c^ Exon 17 (p = 0.03)^c^ Exon 51 (p = 0.02)^c^ (lower risk) Exon 52 (p = 0.05)^c^ (lower risk)		Very low (indirectness; estimates for DMD and BMD; small sample size)	
Kajimoto et al. (2006) [JP] [25]	ACE inhibitors (cilazapril or enalapril)	LVEDD (mm)	Descriptive (one-way repeated measures ANOVA)	*Mean (SD) across 3 years of follow-up* 4.8 (0.6) mm (baseline) vs. 5.3 (0.8) mm (end of follow-up), p < 0.05	Low	Very low (indirectness; estimates for DMD, FCMD, and EDMD)	Very low
		LVEDD (Z-score)		*Mean (SD) Z-score across 3 years of follow-up* 0.5 (0.8) (baseline) vs. 0.8 (0.9) (end of follow-up), p < 0.05			
	ACE inhibitors (cilazapril or enalapril) and β-blockers (carvedilol)	HR (bpm)		*Mean (SD) across 2 years of follow-up* 100 (10) bpm (baseline) vs. 82 (8) bpm (end of follow-up), p < 0.05			
		FS (%)		*Mean (SD) across 2 years of follow-up* 16% (6%) (baseline) vs. 21% (5%) (end of follow-up), p < 0.05			
Kelley et al. (2022) [*] [26]	β-blockers (agents NR)	LVEF (%)	Regression analysis (generalized additive mixed model)	β (main effect): -6.88, SE: 1.57, T-value: -4.38, p < 0.001	Low	–	Low
	Glucocorticoids (agents NR)			β (first order smooths): 0.53, SE: 9, F-value: 0.38, p < 0.01		–	
Kim et al. (2017) [US] [27]	Glucocorticoids (DFZ, PDN, or PRED)	Cardiomyopathy (FS < 28% or EF < 55%)^a^	Regression analysis (Cox proportional hazards model)	HR (treated early vs. untreated): 2.1, 95% CI: 1.2 to 3.5, p < 0.01 HR (treated early vs. treated late): 2.1, 95% CI: 1.2 to 3.5, p = 0.01	Low	–	Low
Koeks et al. (2017) [*] [28]	Glucocorticoids (DFZ, PDN, or PRED)	Cardiomyopathy (definition NR)	Descriptive (χ^2^ test)	*Prevalence of cardiomyopathy at end of follow-up in patients* ≥ *20 years of age* 42% (treated) vs. 62% (previously treated) vs. 60% (never treated), p = 0.0035	Low	–	Low
Kwon et al. (2012) [KR] [29]	ACE inhibitors (enalapril) or β-blockers (carvedilol)	FS (%)	Descriptive (Wilcoxon signed-rank test)	*Mean (SD) after 20.1 months of follow-up* 26.1% (1.7%) (baseline) vs. 27.6% (3.7%) (end of follow-up), p = 0.046	High	Moderate (indirectness; estimates for DMD and BMD)	Moderate
		LVESD (mm/m^2^)		*Mean (SD) across 20.1 months of follow-up* 25.8 (5.7) mm/m^2^ (baseline) vs. 24.1 (4.5) mm/m^2^ (end of follow-up), p = 0.023			
		LVFW systolic myocardial velocity (cm/sec)		*Mean (SD) across 20.1 months of follow-up* 9.1 (2.4) cm/sec (baseline) vs. 7.5 (2.0) cm/sec (end of follow-up), p = 0.005			
Markham et al. (2005) [US] [31]	Glucocorticoids (DFZ or PDN)	Ventricular dysfunction (FS < 28%)	Regression analysis (logistic model)	OR (treated vs. untreated, age 3 to 10 years): 4.4, p = 0.02 OR (treated vs. untreated, age 11 to 21 years): 15.2, p = 0.01	Low	–	Low
Markham et al. (2008) [US] [30]	Glucocorticoids (DFZ or PDN)	Ventricular dysfunction (FS < 28%)	Kaplan–Meier (log-rank test)	Survival functions (treated vs. untreated): p = 0.005	Low	Very low (small sample size)	Very low
			Regression analysis (Cox proportional hazards model)	HR (treated vs. untreated): 0.15, SE: 0.124, 95% CI: 0.03 to 0.74, p = 0.019			
		LVEDD (cm)	Descriptive (Student’s *t*-test)	*Mean (SD) at final echocardiographic measure* 4.2 (0.4) cm (treated) vs. 4.6 (0.9) cm (untreated), p = 0.02			
		FS (%)		*Mean (SD) at final echocardiographic measure* 34% (4%) (treated) vs. 26% (3.6%) (untreated), p < 0.001			
		mWS		*Mean (SD) at final echocardiographic measure* 55 (16) (treated) vs. 73 (31) (untreated), p < 0.020			
		VCFc		*Mean (SD) at final echocardiographic measure* 1.10 (0.22) (treated) vs. 0.87 (0.14) (untreated), p = 0.002			
		VCFc for the given wall stress		*Mean (SD) at final echocardiographic measure* 0.16 (0.16) (treated) vs. -0.10 (0.16) (untreated), p = 0.002			
Matsumura et al. (2010) [JP] [32]	β-blockers (carvedilol)	Composite endpoint (death, deterioration of heart failure and severe arrhythmia)	Regression analysis (Cox proportional hazards model)	HR (treated vs. untreated): 7.181, p = 0.003	Low	–	Low
		HR (bpm)	Descriptive (Student’s *t*-test)	*Mean (SD) HR in patients experiencing the composite endpoint* 89.8 (17.2) bpm (treated) and 80.9 (8.6) bpm (untreated), p = 0.036		–	
Mavrogeni et al. (2009) [GR] [33]	Glucocorticoids (DFZ)	LVEDV (ml)	Descriptive (Student’s *t*-test)	*Median (range) at end of follow-up* 90 (80 to 105) ml (treated) vs. 95 (75 to 120) ml (untreated), p < 0.05	Low	Very low (small sample size)	Very low
		LVEF (%)		*Median (range) at end of follow-up* 53% (51% to 57%) (treated) vs. 48% (42% to 51%) (untreated), p < 0.001			
Nagai et al. (2020) [JP] [34]	Genotypes (RR, RX, and XX; and ACTN3 null or positive genotype)	Cardiac dysfunction (LVEF < 53%)	Kaplan–Meier (log-rank test)	*Median cardiac dysfunction-free survival* 13.4 years (ACTN3 null genotype) vs. 15.3 years (ACTN3 positive genotype), p = 0.041	Low	–	Low
			Mantel–Haenszel test	HR (ACTN3 null genotype vs. ACTN3 positive genotype): 2.78, 95% CI: 1.04 to 7.44, p < 0.05		–	
		LV dilation (LVEDD > 55 mm)	Kaplan–Meier (log-rank test)	Survival functions (RR vs. RX vs. XX): p = 0.023 Survival functions (ACTN3 null genotype vs. ACTN3 positive genotype): p < 0.007		–	
			Mantel–Haenszel test	HR (ACTN3 null genotype vs. ACTN3 positive genotype): 9.04, 95% CI: 1.77 to 46.20, p < 0.05		–	
Porcher et al. (2021) [FR] [35]	ACE inhibitors (perindopril, enalapril, ramipril, or lisinopril)	Hospitalization for heart failure	Regression analysis (Cox proportional hazards model)	HR (treated vs. untreated): 0.50, 95% CI: 0.26–0.99, p < 0.05 • Adjusted HR (treated vs. untreated): 0.16, 95% CI: 0.04–0.62, p < 0.05 HR propensity score-based analysis (treated vs. untreated): 0.37, 95% CI: 0.20–0.68, p < 0.05	Low	–	Low
Posner et al. (2016) [US] [36]	Subjective arm and leg strength	FS (%)	Correlation analysis (Spearman’s correlation coefficient [ρ])	Correlation between subjective arm strength and FS: ρ: 0.47, p = 0.004 Correlation between subjective leg strength and FS: ρ: 0.48, p = 0.003	Low	–	Low
	Total QMT		Regression analysis (generalized least square model)	Correlation between total QMT and FS: coefficient: NR, p = 0.01		–	
Raman et al. (2015) [US] [37]	Mineralocorticoid receptor antagonists (eplerenone)	LVCS (%)	Descriptive (Student’s *t*-test or Wilcoxon signed-rank test)	*Median (IQR) change after 12 months of follow-up* 1.0% (0.3 to -2.2) (treated) vs. 2.2% (1.3 to -3.1) (untreated), p = 0.020	High	–	High
		LVEF (%)		*Median (IQR) change after 12 months of follow-up* -1.8% (-2.9 to 6.0) (treated) vs. -3.7% (-10.8 to 1.0) (untreated), p = 0.032		–	
		LVESV (ml)		*Mean (SD) change after 12 months of follow-up* -1.64 (7.89) ml (treated) vs. 4.07 (8.25) ml (untreated), p = 0.034		–	
Schram et al. (2013) [CA] [38]	Glucocorticoids (DFZ or PDN)	HF-related mortality	Descriptive (χ^2^ test)	*Proportion dying from HF* 0% (0 of 63) (treated) vs. 22% (5 of 23) (untreated), p = 0.0010	Low	–	Low
		LVEF (%)	Descriptive (Student’s t-test or Mann–Whitney *U* test)	*Mean (SD) at end of follow-up* 53% (7%) (treated) vs. 42% (13%) (untreated), p = 0.0008		–	
				*Mean annual rate of change across follow-up* -0.43% (treated) vs. -1.09% (untreated), p = 0.0101		–	
		FS (%)		*Mean (SD) at end of follow-up* 29% (5%) (treated) vs. 23% (7%) (untreated), p = 0.0043		–	
				*Mean annual rate of change across follow-up* -0.32% (treated) vs. -0.65% (untreated), p = 0.0025		–	
		LVEDD (mm)		*Mean (SD) at end of follow-up* 46 (7) mm (treated) vs. 51 (11) mm (untreated), p = 0.0341		–	
				*Mean annual rate of change across follow-up* 0.47 (treated) vs. 0.92 (untreated), p = 0.0105		–	
		LVESD (mm)		*Mean (SD) at end of follow-up* 32 (7) mm (treated) vs. 40 (12) mm (untreated), p = 0.0146		–	
		Cardiomyopathy (EF < 45%)	Kaplan–Meier (log-rank test)	Survival functions (treated vs. untreated): p < 0.001		–	
			Regression analysis (Cox proportional hazards model)	HR (treated early vs. untreated): 0.38, 95% CI: 0.16 to 0.90, p = 0.0270		–	
Servais et al. (2015) [FR] [39]	Deletions treatable by exon 53 skipping (DMD-53)	LVEF (%)	Descriptive (Wilcoxon signed-rank test)	*Mean (SD) at end of follow-up* 50.3% (9.1%) (DMD-53) vs. 63.6% (7.4%) (DMD all-non-53), p = 0.018 50.3% (9.1%) (DMD-53) vs. 66.7% (5.0%) (DMD del-non-53), p = 0.028	Very low	–	Very low
Silva et al. (2017) [BR] [40]	ACE inhibitors (enalapril)	MF (% of LV mass)	Descriptive (Mann–Whitney *U* test)	*Mean (SD) change after 24 months of follow-up* 3.1% (7.4%) (treated) vs. 10.0% (6.2%) (untreated), p = 0.001	High	Moderate (indirectness; estimates for DMD and BMD)	Moderate
			Regression analysis (linear model)	β (treatment vs. no treatment): -4.51, (SE: 2.11), p = 0.04			
		LVEF (%)		β (treatment vs. no treatment): -3.35, (SE: 1.95), p = 0.09			
Silversides et al. (2003) [CA] [41]	Glucocorticoids (DFZ)	LVESD (mm)	Descriptive (Student’s *t*-test or Fisher’s exact test)	*Mean (SD) at end of follow-up* 30 (6) mm (treated) vs. 37 (8) mm (untreated), p = 0.020	Low	–	Low
		LVEF (%)		*Proportion with left LVEF* < *45% at end of follow-up* 5% (1 of 21) (treated) vs. 58% (7 of 12) (untreated), p = 0.001		–	
		FS (%)		*Mean (SD) at end of follow-up* 33% (7%) (treated) vs. 21% (8%) (untreated), p = 0.002		–	
		Systolic blood pressure (mm Hg)		*Mean (SD) at end of follow-up* 106 (7) mm Hg (treated) vs. 112 (5) mm Hg (untreated), p = 0.040		–	
Tandon et al. (2015) [US] [42]	Glucocorticoids (DFZ and/or PDN)	LVEF (%)	Regression analysis (linear mixed-effects model)	β (treatment duration): -0.43% (SE: 0.11%), p < 0.0001	Low	–	Low
Trucco et al. (2020) [UK] [43]	Glucocorticoids (DFZ or PDN)	FS (%)	Regression analysis (mixed-effects model)	*Mean (95% CI) annual rate of decline* 0.53% (0.40% to 0.67%) (treated) and 1.17% (0.79% to 1.55%) (untreated) (p < 0.01)	Low	–	Low
		Cardiomyopathy (FS < 28%)	Regression analysis (Cox proportional hazards model)	HR (untreated vs. treated): 2.2, 95% CI: 1.1 to 4.6, p < 0.05		–	
Viollet et al. (2012) [US] [44]	ACE inhibitors (lisinopril) and/or β-blockers (metoprolol or atenolol)	EF (%)	Descriptive (Student’s *t*-test)	*Mean (SD) before initiation of ACE inhibitor and/or BB therapy* 54% (8.1%) (months -12 to -6) and 47% (7.4%) (months -6 to 0), p = 0.006 *Mean (SD) with ACE inhibitor treatment* 47% (6.1%) (months 0 to 6) and 52% (8.4%) (months 6 to 12), p = 0.011 *Mean (SD) with ACE inhibitor and β-blocker treatment* 46% (10%) (months 0 to 6) and 50% (11%) (months 6 to 12), p = 0.001	Low	–	Low
Yamamoto et al. (2018) [JP] [45]	Mutations in the Dp116 coding region (vs. other dystrophin isoform deficiencies)	Cardiac dysfunction (LVEF < 53%)	Kaplan–Meier (log-rank test)	Survival functions (Dp116 vs. other): p = 0.022	Low	–	Low
Zhang et al. (2015) [CN] [46]	Glucocorticoids (agents NR)	SRS	Descriptive (Student’s *t*-test)	*Mean (SD) score* Age 7 years: 4.40 (1.58) (before) vs. 8.20 (4.59) (after), p < 0.01 Age 8 years: 4.00 (1.75) (before) vs. 6.00 (7.00) (after), p < 0.01 Age 9 years: 3.55 (1.75) (before) vs. 7.27 (5.27) (after), p < 0.01 Age 10 years: 6.30 (2.45) (before) vs. 10.00 (3.68) (after), p < 0.01	Low	–	Low

Angiotensin receptor blocker (ARB). Angiotensin-converting enzyme (ACE). Becker muscular dystrophy (BMD). Brazil (BR). Canada (CA). China (CN). Confidence interval (CI). Deflazacort (DFZ). Dilated cardiomyopathy (DCM). Duchenne muscular dystrophy (DMD). Ejection fraction (EF). Fractional shortening (FS). France (FR). Greece (GR). Hazard ratio (HR). Heart failure (HF). Heart rate (HR). Inter-quartile range (IQR). Japan (JP). Left ventricular (LV). Left ventricular circumferential strain (LVCS). Left ventricular ejection fraction (LVEF). Left ventricular end-diastolic diameter (LVEDD). Left ventricular end-diastolic volume (LVEDV). Left ventricular end-systolic diameter (LVESD). Left ventricular end-systolic volume (LVESV). Left ventricular free wall (LVFW). Left ventricular mass (LVM). Meridional wall stress (mWS). Myocardial fibrosis (MF). Myocardial performance index (MPI). Not applicable (NA). Not reported (NR). Odds ratio (OR). Prednisolone (PRED). Prednisone (PDN). Quantitative muscle testing (QMT). Randomized controlled trial (RCT). South Korea (KR). Standard deviation (SD). Standard error (SE). Summed rest score (SRS). United Kingdom (UK). United States of America (US). Velocity of circumferential fiber shortening (VCFc)

^*^Multi-national (see article for details)

^a^If neither FS nor EF were reported, then FS was calculated using M-Mode data of LVEDD and LVESD

^b^ > 2 Z-scores from normal values for body surface area

^c^Estimated coefficient not reported

We identified one uncontrolled clinical trial, Kwon et al. [29], describing the efficacy of ACE inhibitors (enalapril) or β-blockers (carvedilol) in 23 Korean patients (22 with DMD and one with BMD; mean age: 13 years, range not reported). After 20.1 months of follow-up, fractional shortening (FS), left ventricular end-systolic diameter (LVESD), and left ventricular free wall (LVFW) systolic myocardial velocity were significantly improved compared with baseline values in patients treated with either enalapril or carvedilol (criteria for initiation not reported) (all p ≤ 0.046).

Looking at results from identified observational research, significantly improved LVEF was reported by Aikawa et al. [14] in their study of 34 Japanese patients (21 with DMD and 13 with BMD) treated with ACE inhibitors (cilazapril and enalapril) (initiated at different LVEF levels), in some cases in combination with β-blockers (bisoprolol) and/or ARBs (agents not reported); Jefferies et al. [24] in 69 US patients (62 with DMD and seven with BMD) treated with ACE inhibitors (enalapril, captopril, and lisinopril) (initiated at an LVEF ≥ 55% or evidence of left ventricular dilation) and/or β-blockers (carvedilol and metoprolol); and Kelley et al. [26] in a multi-national cohort comprising of 147 patients with DMD treated with β-blockers (agents not reported) (criteria for initiation not reported) (in some cases in combination with ACE inhibitors, ARBs, diuretics agents, anti-arrhythmics, and/or inotropes [agents not reported]).

We identified one retrospective cohort study, Porcher et al. [35], examining the impact of prophylactic use of ACE inhibitors (perindopril, enalapril, ramipril, or lisinopril) on the risk of hospitalization for heart failure among 576 French patients with DMD with normal left ventricular function. Compared with no treatment, ACE inhibitors (initiated at a LVEF ≥ 55%) were associated with a significant risk reduction (HR: 0.50, 95% CI: 0.26 to 0.99, p < 0.05; adjusted HR: 0.16, 95% CI: 0.04 to 0.62, p < 0.05; and HR propensity score-based analysis: 0.37, 95% CI: 0.20–0.68, p < 0.05).

Viollet et al. [44] showed ejection fraction (EF) improvement compared to baseline 12 months before initiation of therapy in 42 patients receiving either ACE inhibitor (lisinopril) only, or ACE inhibitor plus a β-blocker (metoprolol or atenolol) (p < 0.0001); however, ACE inhibitor plus β-blocker was not superior than ACE inhibitor alone.

Further evidence of benefits of cardiac medications in DMD include improved FS [25]; HR [25, 32]; left ventricular end-diastolic diameter (LVEDD) [24, 25]; LVEDD (Z-score) [25]; left ventricular end-diastolic volume (LVEDV) [17]; LVESD [29]; LVESV [17]; LVFW systolic myocardial velocity (cm/sec) [29]; left ventricular mass (LVM) [17]; left ventricular myocardial performance index (MPI) [24]; left ventricular sphericity index [24]; and death, deterioration of heart failure and severe arrhythmia (composite endpoint) [32]. One study also examined the impact of the timing of initiation of ACE inhibitors (cilazapril and enalapril) in terms of LVEF in patients with DMD or BMD [14].

#### DMD mutations

We identified four observational studies reporting evidence of effects of *DMD* mutations on cardiac disease in DMD (Table 2). Specifically, in a retrospective cohort study of 69 patients with DMD and BMD (of which 47 had genetic analysis of their deoxyribonucleic acid [DNA]), Jefferies et al. [24] investigated the association between *DMD* mutations and age at onset of cardiomyopathy (defined as EF < 55% or left ventricular dilation). Mutations involving exons 12, 14, 15, 16, and 17 (type not reported) were all shown to be associated with onset of cardiomyopathy, and exons 51 and 52 appeared to be associated with lower risk of cardiac involvement. In the retrospective cohort study by Yamamoto et al. [45], encompassing 181 Japanese children and adults with DMD, patients with mutations in the Dp116 coding region were found to have a significantly longer cardiac dysfunction-free survival than those with other dystrophin isoform deficiencies (p = 0.022). Moreover, Cirino et al. [19] found that exons 45, 46, 47, 48, 49, 50, and 52 were associated with early left ventricular systolic dysfunction (all p < 0.044) among 40 Brazilian patients with DMD. Finally, in a case series by Servais et al. [39], comprising of 35 non-ambulatory French patients with DMD, LVEF was estimated at 50.3% in patients with deletions treatable by exon 53 skipping (DMD-53), 63.6% in patients with mutations not treatable by exon 53 skipping (DMD all-non-53), and 66.7% in patients with deletions not treatable by exon 53 skipping (DMD del-non-53) at end of follow-up (DMD-53 vs. DMD all-non-53: p = 0.018; DMD-53 vs. DMD del-non-53: p = 0.028).

#### Genetic modifiers

We identified two observational studies reporting evidence of effects of genetic modifiers on cardiac disease in DMD (Table 2). Specifically, Barp et al. [16] studied genetic modifiers for dilated cardiomyopathy in a sample of 178 Italians with DMD and found that patients with the *LTBP4* rs10880 CC/CT genotype had a higher risk of dilated cardiomyopathy compared with the TT genotype (p < 0.027). Moreover, Nagai et al. [34] described cardiac dysfunction (defined as LVEF < 53%) and left ventricular dilation (defined as LVEDD > 55 mm) by genotype in 77 Japanese patients with DMD. Median cardiac dysfunction-free survival was 13.4 years and 15.3 years (p = 0.041) in patients with the *ACTN3* null genotype and *ACTN3* positive genotype, respectively (HR: 2.78, 95% CI: 1.04 to 7.44, p < 0.05). The left ventricular dilation-free survival rate was different between patients with the RR, RX, and XX genotypes (p = 0.023) and lower in patients with the *ACTN3* null genotype compared with the *ACTN3* positive genotype (HR: 9.04, 95% CI = 1.77 to 46.20, p < 0.05).

#### Glucocorticoid exposure

We identified six observational studies reporting evidence of benefits of glucocorticoids on LVEF in patients with DMD (Table 2). Specifically, Biggar et al. [18] found the proportion of patients with LVEF < 45% at 18 years of age to be lower among those receiving glucocorticoid therapy (deflazacort) compared with no glucocorticoid therapy (10%, vs. 58%, p < 0.001); Mavrogeni et al. [33] estimated the median LVEF at end of follow-up (duration not reported) at 53% and 48% in patients with and without glucocorticoid treatment (deflazacort), respectively (p < 0.001); Kelley et al. [26] reported of improved LVEF in patients treated with glucocorticoids (agents not reported); Silversides et al. [41] estimated the proportion of patients with LVEF < 45% at end of follow-up (duration not reported) at 5% with glucocorticoid therapy (deflazacort) and 58% without glucocorticoid therapy (p = 0.001); and Schram et al. [38] estimated the mean annual rate of change in LVEF across follow-up at -0.43% for patients treated with glucocorticoids (deflazacort or prednisone) and -1.09% for those with no glucocorticoid treatment (p = 0.0101). Additionally, Tandon et al. [42] found a significant negative association between duration of glucocorticoid therapy (deflazacort or prednisone) and LVEF in a retrospective cohort study of 98 US patients with DMD. Specifically, an increased glucocorticoid treatment duration was associated with an LVEF decline of 0.43% per year of treatment (p < 0.0001).

We identified six observational studies reporting evidence of benefits of glucocorticoids on FS in patients with DMD. Specifically, Biggar et al. [18] estimated the mean FS at 18 years of age at 33% and 21% in patients with and without glucocorticoid treatment (deflazacort), respectively (p < 0.002); Houde et al. [23] estimated the mean FS at end of follow-up (duration not reported) at 30.8% in participants treated with glucocorticoids (deflazacort) (in some cases in combination with ACE inhibitors [agents not reported]) compared with 26.6% in those not receiving glucocorticoid therapy (p < 0.05); Markham et al. [30] found the mean FS to be higher in patients with DMD treated with glucocorticoids (deflazacort or prednisone) compared to those who were not treated (34% vs 26%, p < 0.001); Schram et al. [38] estimated the mean FS at end of follow-up at 29% for patients treated with glucocorticoids (deflazacort or prednisone) and 23% for untreated participants (p = 0.0043); Silversides et al. [41] estimated the mean FS at 33% and 21% with and without glucocorticoid treatment (deflazacort), respectively (p = 0.002); and Trucco et al. [43] estimated the mean annual rate of decline at 0.53% in those treated with glucocorticoids (deflazacort or prednisone) and at 1.17% in patients not treated (p < 0.01). Additionally, in two separate retrospective cohort studies, Markham et al. [30, 31] evaluated the frequency of ventricular dysfunction (defined as FS < 28%) after glucocorticoid treatment. The authors found that those receiving glucocorticoids (deflazacort or prednisone) had a significantly lower risk of ventricular dysfunction compared to untreated patients (all p ≤ 0.02).

We identified five observational studies describing the effects of glucocorticoids on cardiomyopathy outcomes. Specifically, Houde et al. [23] found the proportion of participants with dilated cardiomyopathy (defined as FS < 28% or LVEDD > 95th percentile) to be lower among those treated with deflazacort (in some cases in combination with ACE inhibitors [agents not reported]) than those who were untreated (32% vs. 58%, p < 0.05). Similarly, in a multi-national cohort comprising of 5,345 patients with DMD, Koeks et al. [28] reported that 42% and 60% of patients ≥ 20 years of age with and without glucocorticoid exposure (deflazacort, prednisone, or prednisolone), respectively, had evidence of cardiomyopathy at end of follow-up (p = 0.0035). The prevalence of cardiomyopathy among patients previously treated with glucocorticoids was 62%. Moreover, Schram et al. [38] investigated the risk of cardiomyopathy (defined as EF < 45%) in 86 Canadian patients with DMD and found glucocorticoids (deflazacort or prednisone) to have a protective effect (HR: 0.38, 95% CI: 0.16 to 0.90, p = 0.0270). In line with these results, Trucco et al. [43] found that patients not treated with glucocorticoids (deflazacort or prednisone) had a higher risk of cardiomyopathy (defined as FS < 28%) compared with their treated counterparts (neither group exposed to any cardiac medication) (HR: 2.2, 95% CI: 1.1 to 4.6, p < 0.05). In the study by Barber et al. [15], involving 462 US participants with DMD, a significant inverse association was observed between glucocorticoid duration and timing of onset of cardiomyopathy (defined as FS < 28% or EF < 55%). Specifically, the probability of developing cardiomyopathy decreased by 4% for every year of treatment with glucocorticoids (p < 0.001). In contrast, the study by Kim et al. [27], comprising of 660 US patients with DMD, reported an increased risk of cardiomyopathy (defined as FS < 28% or EF < 55%) in participants treated early with glucocorticoids compared to those who were untreated (HR: 2.1, 95% CI: 1.2 to 3.5, p < 0.01), as well as in those treated early vs. late (HR: 2.1, 95% CI: 1.2 to 3.5, p = 0.01).

We identified one observational study reporting evidence of effects of glucocorticoids on heart failure (HF)-related mortality. Specifically, among 86 Canadians with DMD, Schram et al. [38] found that the proportion of patients who died from HF-related causes was 0% in those treated with glucocorticoids (deflazacort or prednisone) and 22% in untreated patients (p = 0.0010) (all of whom also received cardiac medication).

Further evidence of benefits of glucocorticoids in DMD include improved EF [23]; LVEDD [30, 38]; LVEDV [33]; LVESD [38, 41]; meridional wall stress (mWS) [30]; systolic blood pressure [41]; summed rest score (SRS) [46]; velocity of circumferential fiber shortening (VCFc) [30]; and ventricular dysfunction [31].

#### Muscle strength

We identified one retrospective cohort study examining the relationship between muscle strength and FS in patients with DMD (Table 2). Specifically, Posner et al. [36] presented evidence of significant correlations between subjective arm and leg strength and total quantitative muscle testing, respectively, and FS (p ≤ 0.01), among 77 US children and adults with DMD.

#### Ventilation support

We identified one retrospective cohort study describing an effect of mechanical ventilation on LVEF (Table 2). Specifically, Fayssoil et al. [22] reported a significant inverse relationship between full-time mechanical ventilation and annual rate of LVEF decline among 101 French adults with DMD (p = 0.012).

### Rating of the certainty of the evidence

Per the manual of GRADE, we initially attributed included RCTs a high rating, observational studies a low rating, and case reports a very low rating. Next, we downgraded the rating for Aikawa et al. [14], Jefferies et al. [24], and Kajimoto et al. [25], Kwon et al. [29], and Silva et al. [40] due to indirectness (as the studies also included patients with diseases other than DMD); Duboc et al. [21] and Dittrich et al. [20] due to inconsistency of results; and Jefferies et al. [24], Markham et al. [30], Mavrogeni et al. [33], and Cirino et al. [19] due to small sample sizes (overall and/or by examined strata). Finally, we provided an overall rating of the certainty of the evidence of each study (Table 2).

## Discussion

Across the past couple of decades, the successful dissemination of a coordinated, multidisciplinary approach to the clinical management of DMD has realized remarkable improvements to prognosis. Yet, as patients walk and live longer, new challenges have emerged, especially for cardiologists. Indeed, the development of therapeutic strategies responding to the additional strain on the heart associated with prolonged ambulation, as well as increased life-expectancy, has emerged as one of the most pressing clinical issues in this heavily burdened patient population. A key component to this effort, relevant to both clinical practice and research, is an increased understanding of sources of cardiac heterogeneity. To that end, in this systematic literature review, encompassing a total of 33 studies involving 9,232 patients from 11 countries, we synthesized and graded the body of evidence of predictors of cardiac disease in DMD.

Exposure to cardiac medication, including ACE inhibitors, β-blockers, and mineralocorticoid receptor antagonists, has been shown to have a significant effect on a wide range of commonly evaluated cardiac outcomes in patients with DMD. However, in many studies, the individual contribution from these pharmacological agents remains to some degree unknown, since they are commonly prescribed in combination. For example, in the study of β-blockers by Kelley et al. [26], some patients were concurrently treated with ACE inhibitors and/or ARBs, diuretics, anti-arrhythmics, and inotropes, and many were receiving glucocorticoids, which also are associated with cardiac disease in DMD (as discussed below). We also found few estimates pertaining to specific features of pharmacological cardiac intervention, such as the comparative effect of different doses or regimens, but one study examined the impact of the timing of initiation of ACE inhibitors in patients with DMD or BMD, reporting of a significant effect only among those treated at LVEF < 55% [14]. Similar negative findings have been more recently reported from a RCT of children with DMD (mean age: 9 years) with normal ventricular function treated with ACE inhibitors and β-blockers for 36 months [47] (which is not surprising given that cardiac dysfunction is not expected at these ages in patients with DMD [8]). Considering the increased importance of cardiac management in DMD following prolonged ambulation and survival, further research is warranted to help understand optimal treatment algorithms of cardiac medication in this patient population, including benefits and harms of prophylactic intervention.

Several identified studies focused on the genotype–phenotype association with dystrophin-deficient cardiomyopathy. Mutations in exons 51 and 52, deletions treatable by exon 53 skipping, and mutations involving the Dp116 coding region, have been shown to have a comparatively protective effect against cardiomyopathy [24, 39, 45]. However, in terms of mutations associated with a higher risk and early onset of cardiac disease, we found some potential inconsistent results. While some authors observed that particularly distal or downstream mutations were associated with early left ventricular systolic dysfunction [19], other authors reported that more proximal or upstream mutations were associated with an early onset of cardiomyopathy [24]. Furthermore, Jeffries et al. [24] found that mutations in exon 52 were protective against cardiomyopathy; while Cirino et al. [19], reported an early onset of left ventricular systolic dysfunction with involvement of this same exon. In addition, other genes than DMD have also been linked to cardiac outcomes and have been mentioned as potential prognostic factors. Particularly, *LTBP4* and *ACTN3* polymorphisms and genotypes have been proposed to be associated with a higher risk of dilated cardiomyopathy [16, 34]. Concerning the interpretation of the synthesized evidence of *DMD* mutations and DMD genetic modifiers, it is important to keep in mind that the field of genetics/genomics in DMD is still advancing. As such, some publications of this topic report results from relatively small pilot studies of low certainty. However, this does not mean that the potential importance of *DMD* mutations and DMD genetic modifiers is low, or that further investigation of *DMD* mutations and DMD genetic modifiers is not warranted. Instead, our synthesis should be viewed as the *current* state of the evidence-base, expected to be amended by future research, through which our understanding and certainty of the evidence of specific genetic factors in DMD is expected to be greatly enhanced.

Glucocorticoids have a significant, positive effect on a wide range of cardiac outcomes in DMD. Yet, similar to cardiac medications (discussed above), little is known of the comparative impact of specific agents or regimens. In most studies, it is also difficult to elicit the effects specific to glucocorticoids, since they are commonly prescribed together with, for example, ACE inhibitors and β-blockers. Interestingly, Kim et al. [27] found that patients treated early with glucocorticoids had worse outcomes than those who remained untreated or treated late. A possible explanation for this finding includes confounding by indication, in which those treated early are clinically different from those not treated or treated late, for example, by being subject to a particularly aggressive disease trajectory (which could trigger early intervention). Nonetheless, the impact of different timings of, or criteria for, treatment initiation on cardiac disease in DMD remains largely unknown and warrants further study.

Fayssoil et al. [22] reported full-time mechanical ventilation support to be significantly associated with more favorable cardiac progression. Although not yet replicated in other samples of patients with DMD, as noted by the authors, these findings are supported by previous research showing that ventilatory support can help increase intrathoracic pressure and thus decrease left ventricular afterload. Yet, it is important to keep in mind that similar to most studies in this review, Fayssoil et al. [22] studied patients also receiving ACE inhibitors, β-blockers, and diuretics. It is therefore not possible to quantify the specific contribution of ventilatory support on cardiac disease based on the reported data.

Our findings have several implications for clinical practice and research. First, understanding predictors of cardiac disease, including phenotypic variability as part of the natural disease evolution, is important for tailoring patient-specific treatment algorithms, as well as to shape expectations of realistic treatment outcomes. Second, evidence of predictors of cardiac disease is critical also to the design RCTs of new pharmaceutical interventions in DMD to ensure adequate internal and external validity. Indeed, pooling patients with vastly different disease trajectories, in particular those exhibiting extreme phenotypes (either protective or detrimental) is likely to produce estimates of treatment effects that are challenging to interpret and difficult to generalize. Third, and last, the data synthesized as part of this review would also be expected to help inform matching algorithms and similar statistical procedures employed to indirectly compare and contextualize evidence obtained from single-arm trials to outcomes observed in natural history studies. This is likely to become increasingly important as the pipeline of new experimental treatments, including gene therapies, is reaching testing in human clinical trials in the coming decade [48].

Our study is subject to a few limitations. First, to ensure relevance to current clinical care practices, we limited the search to account for records published from the calendar year 2000. Although unlikely, we might thus have missed some data applicable to the review topic. Second, it is important to emphasize that we were unable (based on the reported evidence) to compare the impact of specific predictors of cardiac disease, for example genetic versus therapeutic effects. That being said, from our review, it is clear that such an analysis would be quite challenging to perform because of the number of potential predictors the typical patient with DMD simultaneously is subject to at a given time (e.g., genetic modifier, cardiac medication, and glucocorticoids). Large studies of predictors of cardiac disease in DMD might help delineate some of the individual effects; yet, from an epidemiological point of view, eliciting the causal effects of individual predictors is likely to remain a challenge, in particular for less common genetic expressions. Third, in concordance with the review objective, we did not account for predictors of progression of myocardial disease based on cardiovascular assessments (e.g., the relationship between myocardial fibrosis, MRI, and/or blood biomarkers, respectively, and the development of systolic dysfunction and heart failure), since this would necessitate an independent search strategy encompassing dedicated search criteria, and also considering the scale of the current review (as adding numerous additional factors, identified via a separate protocol, would greatly expand the scope and complexity of the study). Identifying this evidence is, however, an important topic for future research. Fourth, and last, we did not recognize and include certain factors known to impact cardiac health and function more generally (e.g., exercise and obesity), as our review, by design, focused on evidence derived from populations of patients with DMD.

## Conclusions

Several sources of cardiac disease heterogeneity have been delineated in patients with DMD, including cardiac medication (moderate- to high-quality evidence), *DMD* mutations (low/very low-quality evidence), DMD genetic modifiers (low-quality evidence), glucocorticoid exposure (high-quality evidence), muscle strength (low-quality evidence), and ventilation support (low-quality evidence). Yet, little is known of the contribution of non-pharmacological interventions, as well as the impact of different criteria for initiation of specific treatments. Our findings help raise awareness of prevailing unmet needs, shape expectations of treatment outcomes, and inform the design of future research.

## Supplementary Information

**Additional file 1**. **eTable 1:** Search terms for MEDLINE ALL (including MEDLINE daily, MEDLINE ePub ahead of print, MEDLINE In-Process). **eTable 2:** Search terms for Embase. **eTable 3:** Search terms for the Cochrane Database of Systematic Reviews.

## Data Availability

All data generated or analysed during this study are included in this published article [and its supplementary information files].
